# Results of a proof of concept, double-blind, randomized trial of a second generation antisense oligonucleotide targeting high-sensitivity C-reactive protein (hs-CRP) in rheumatoid arthritis

**DOI:** 10.1186/s13075-015-0578-5

**Published:** 2015-03-19

**Authors:** Marshelle S Warren, Steven G Hughes, Walter Singleton, Mason Yamashita, Mark C Genovese

**Affiliations:** Isis Pharmaceuticals, Inc, 2855 Gazelle Court, Carlsbad, CA 92010 USA; Stanford University, 1000 Welch Rd Palo, Alto, CA 94304 USA

## Abstract

**Introduction:**

This randomized, double-blind, phase II study evaluated the pharmacodynamics, safety and tolerability of ISIS 329993 (ISIS-CRP_Rx_), an antisense oligonucleotide, in patients with active rheumatoid arthritis (RA).

**Methods:**

Patients with active RA of at least six months duration were randomized into three cohorts to receive ISIS-CRP_Rx_ (100 mg, 200 mg or 400 mg) or placebo (3 active:1 placebo within each cohort) via subcutaneous (SC) injection on Days 1, 3, 5 and 8 and then once weekly for the next 11 weeks. The effects of study treatment on high-sensitivity C-reactive protein (hs-CRP) level were evaluated. An exploratory analysis on disease activity was assessed via the American College of Rheumatology 20% improvement criteria (ACR20). Safety was evaluated via adverse events and laboratory measures.

**Results:**

Fifty-one patients received one of the following treatments: ISIS-CRP_Rx_ 100 mg, n = 12; 200 mg, n = 13, 400 mg, n = 14; placebo n = 12. In the ISIS-CRP_Rx_ treatment groups there were dose-dependent reductions in hs-CRP. At Day 36 the mean percent change from baseline was: placebo: −14.4%; ISIS-CRP_Rx_ 100 mg: −19.5%; 200 mg: −56.6% and 400 mg: −76.7%, (*P* = 0.0015 placebo compared to 400 mg). There were no differences between treatment groups and placebo in the ACR20 at Day 36 or Day 92. There were no serious infections and no elevations in liver function tests, lipids, creatinine or other lab abnormalities related to ISIS-CRP_Rx_.

**Conclusions:**

In this study, ISIS-CRP_Rx_ selectively reduced hs-CRP in a dose-dependent manner, and was well-tolerated in patients with RA. Its utility as a therapy in RA remains unclear.

**Trial registration:**

Clinicaltrials.gov NCT01414101. Registered 21 July 2011.

**Electronic supplementary material:**

The online version of this article (doi:10.1186/s13075-015-0578-5) contains supplementary material, which is available to authorized users.

## Introduction

Studies of patients with rheumatoid arthritis (RA) document a correlation between C-reactive protein (CRP) blood concentration and worsening of RA symptoms. Inflammation in RA is closely related to the production of CRP and proinflammatory cytokines [1]. Levels of CRP correlate closely with changes in inflammation/disease activity, radiological damage and progression, and functional disability [1]. Although CRP is considered to be a marker of inflammation in RA, it may also function in the promotion of inflammation through complement activation [2]. CRP, and in particular the high-sensitivity CRP (hs-CRP) assay, has been shown to be more closely associated with disease activity variables than erythrocyte sedimentation rate (ESR) [3].

ISIS 329993 (ISIS-CRP_Rx_) is an antisense drug targeted to human CRP. ISIS-CRP_Rx_ has been tested in a rodent model of RA (that is, CRP transgenic mice with collagen-induced arthritis) and was shown to improve the clinical signs of arthritis [4]. Further, in a previously conducted clinical study in healthy human volunteers (N = 8), whose blood hs-CRP levels ranged from ≥2 to 10 mg/L on two qualifying examinations within a two-week period, treatment with ISIS-CRP_Rx_ achieved significant hs-CRP lowering [4]. The next step in the clinical development process was to determine if ISIS-CRP_Rx_ would be effective in reducing hs-CRP in patients with a chronic inflammatory disease, RA.

The objectives of this clinical study were to evaluate the pharmacodynamics, safety and tolerability of ISIS-CRP_Rx_ in patients with active RA.

## Methods

This Phase II, international, multi-center, double-blind, placebo-controlled, parallel group study in patients with RA was conducted at three sites in Canada and ten sites in Russia with enrollment beginning on 3 October 2011 and ending on 18 December 2012. The Institutional Review Board at each site approved the study protocol and the informed consent (see Additional file 1 for details of the ethical bodies). This study was performed in accordance with globally accepted standards of Good Clinical Practice (as defined in the International Conference on Harmonisation E6 Guidelines for Good Clinical Practice, 1 May 1996), and in agreement with the Declaration of Helsinki and in keeping with local regulations. Prior to screening, all subjects provided written informed consent. The trial was registered with clinicaltrials.gov (www.clinicaltrials.gov) [Identification number: NCT01414101].

### Patients

Patients (18 to 75 years of age) with active RA, as defined by the American Rheumatism Association 1987 revised criteria [5], for at least six months, and a function Class I-III classified according to the American College of Rheumatology 1991 criteria were enrolled [6]. Patients were required to have hs-CRP of ≥5 mg/L at screening (upper limit of normal >3 mg/L) with at least six swollen joints and at least six tender joints, based on a 28-joint count. All patients had received at least three months of methotrexate therapy at a stable dose of ≥10 mg unless they could not tolerate that dose. Methotrexate, at stable dose, was continued throughout the study. Other concomitant disease-modifying antirheumatic drugs (DMARDs), non-steroidal anti-inflammatory drugs (NSAIDs) and prednisone at 10 mg daily or less were stable prior to and during the study.

### Interventions

Eligible patients were randomized in an equal ratio to one of the three dose cohorts (100 mg ISIS-CRP_Rx_ or placebo; 200 mg ISIS-CRP_Rx_ or placebo; or 400 mg ISIS-CRP_Rx_ or placebo). Within each dose cohort, patients were randomized to receive ISIS-CRP_Rx_ or placebo in a 3:1 ratio. Enrolled patients received subcutaneous (SC) injections of ISIS-CRP_Rx_ or placebo on Days 1, 3, 5 and 8 and then once weekly for the next 11 weeks.

### Outcomes

#### Pharmacodynamic

The effects of treatment with ISIS-CRP_Rx_ versus placebo on hs-CRP and other markers of inflammation (for example, ESR, serum amyloid A (SAA), interleukin-6 (IL-6), tumor necrosis factor alpha (TNF-α), tumor necrosis factor receptor 1 (TNF-RI), tumor necrosis factor receptor 2 (TNF-RII), fibrinogen and complement) were evaluated throughout the study. During screening, patients had to have at least two hs-CRP measurements taken at least one week apart and no more than one week prior to initiation of dosing and the average of these values was used as the subjects’ baseline hs-CRP level. Patient hs-CRP levels and other markers of inflammation were assessed during the treatment and post-treatment evaluation period at regular intervals.

#### Exploratory activity in RA

The American College of Rheumatology 20% improvement criteria (ACR20) is a widely accepted index of improvement in RA [7] that refers to an improvement of at least 20% in a composite measure of the disease. ACR20 response criteria were assessed at baseline on Day 1 prior to dosing and on Day 36 and Day 92. Disease activity was also assessed using the Disease Activity Score Activity Score Calculator for Rheumatoid Arthritis using the ESR (DAS28-ESR) [8]. DAS28-ESR criteria were assessed at baseline on Day 1 prior to dosing and on Day 36 and Day 92. Patients were considered to be in remission if they had a score of less than 2.6 on the DAS28-ESR.

#### Safety

Safety was assessed via adverse events (defined as an adverse event that occurred on or after the first dose of treatment) and clinical laboratory parameters throughout the study.

### Statistics

#### Sample size

It was estimated that the standard deviation of the percent change of hs-CRP was approximately 40% based on the results of a Phase I study [9]. With 12 subjects in each treatment group, the study was designed to have approximately 74% power to detect a 45% difference in hs-CRP between the treated group and the placebo group, assuming the treated group would have a 50% reduction in hs-CRP while the control group would have a 5% reduction. An alpha level of 0.05 was utilized.

#### Randomization

Eligible subjects were randomized equally to one of three dose cohorts (ratio = 1:1:1). Each dose cohort was to consist of 16 subjects. Within each dose cohort, subjects were randomized to receive ISIS-CRP_Rx_ or placebo in a 3:1 ratio, respectively. Subjects were centrally randomized prior to study drug administration (ISIS-CRP_Rx_ or placebo) using an automated system.

#### Blinding

The study was blinded with respect to the treatment allocation for subjects, investigators, coordinators and sponsor. The dose cohort was not blinded.

#### Statistical methods

The safety population consisted of all patients who were randomized and received at least one dose of study drug. The per protocol population consisted of all subjects who were randomized and received at least 60% of doses (≥9 doses), completed the Day 92 or early termination evaluations and did not have any clinically significant protocol deviations.

Demographic and baseline characteristic information was summarized descriptively for each treatment group. Pharmacodynamic endpoints were summarized using the per protocol population. The effects of treatment (ISIS-CRP_Rx_ versus placebo) on hs-CRP and other markers of inflammation were evaluated at multiple time points after dosing. Baseline hs-CRP was determined as the average of all of the values during the screening period, prior to dosing on Day 1. In addition, the effects of treatment (ISIS-CRP_Rx_ versus placebo) were evaluated on the proportion of subjects achieving clinical improvement in RA disease activity at Day 36 and Day 92 compared to baseline using the ACR20 response criteria in the per protocol population.

## Results

### Patients

A total of 51 subjects were randomized in this study (12 placebo, 12 ISIS-CRP_Rx_ 100 mg, 13 ISIS-CRP_Rx_ 200 mg and 14 ISIS-CRP_Rx_ 400 mg) and 41 subjects completed the treatment (11 placebo, 11 ISIS-CRP_Rx_ 100 mg, 9 ISIS-CRP_Rx_ 200 mg and 10 ISIS-CRP_Rx_ 400 mg) (Additional file 2). Demographics and baseline characteristics were similar across the treatment groups (Table 1).

**Table 1:** **Baseline demographics and disease characteristics**

**Demographics (Safety Population)**	**Placebo**	**ISIS-CRP**_**Rx**_	**ISIS-CRP**_**Rx**_	**ISIS-CRP**_**Rx**_
		**100 mg**	**200 mg**	**400 mg**
	**Number = 12**	**Number = 12**	**Number = 13**	**Number = 14**
Race				
White, number (%)	12 (100)	12 (100)	13 (100)	14 (100)
Sex				
Female, number (%)	12 (100)	9 (75)	8 (61.5)	13 (92.9)
Age, years, mean (SD)	64.1 (4.25)	56.08 (13.14)	58.69 (9.81)	62.93 (7.97)
Weight, kg, mean (SD)	72.29 (13.39)	74.05 (19.81)	73.75 (16.43)	72.19 (13.39)
BMI, kg/m^2^, mean (SD)	28.36 (4.97)	27.27 (5.04)	25.78 (4.93)	26.84 (3.63)
Duration of RA since 1st diagnosis, years, mean (SD)	18.6 (15.8)	6.3 (8.1)	8.8 (7.8)	18.3 (12.4)
ACR Functional Class, number (%)				
Class I	1 (8.3)	1 (8.3)	0	1 (7.1)
Class II	6 (50.0)	8 (66.7)	7 (53.8)	7 (50.0)
Class III	5 (41.7)	3 (25.0)	6 (46.2)	6 (42.9)
Morning stiffness, minutes, mean (SD)	61.7 (29.3)	98.3 (97.5)	90.4 (59.0)	106.4 (75.9)
**Disease Characteristics (Per Protocol Population)**	**Number = 11**	**Number = 10**	**Number = 8**	**Number = 11**
Westergren ESR, mm/hour, mean (SD)	41.0 (27.7)	47.6 (27.3)	37.4 (14.0)	44.5 (31.1)
hsCRP, mg/L, mean (SD)	22.4 (18.1)	29.7 (29.0)	35.2 (36.4)	19.3 (13.6)
Swollen joint count, mean (SD)	12.5 (5.6)	9.7 (6.4)	9.8 (4.5)	8.3 (2.2)
Tender joint count, mean (SD)	17.6 (8.3)	15.9 (8.0)	14.8 (7.5)	14.1 (5.7)
DAS28-ESR, mean (SD)	6.66 (0.6)	6.36 (1.1)	6.27 (0.7)	6.23 (0.9)
HAQ, mean (SD)	1.67 (0.6)	1.50 (0.7)	1.58 (0.6)	1.73 (0.6)
Patient’s pain assessment, mean (SD)	66.82 (16.4)	54.50 (16.6)	59.75 (13.2)	61.55 (18.4)
Patient’s global assessment of disease activity, mean (SD)	2.64 (0.7)	2.30 (0.7)	2.38 (0.5)	2.45 (0.5)
Physician’s global assessment of disease activity, mean (SD)	2.18 (0.4)	2.10 (0.7)	2.50 (0.5)	2.36 (0.5)

ACR, American College of Rheumatology; BMI, body mass index; DAS28-ESR, disease activity score 28-erythrocyte sedimentation rate; ESR, erythrocyte sedimentation rate; HAQ, Health Assessment Questionnaire; hsCRP, high sensitivity C-reactive protein; percentage calculated as 100 × n/N; SD, standard deviation.

### Medication exposure

The mean number of doses that patients received was similar across treatment groups in the per protocol population (14.2 placebo, 15 ISIS-CRP_Rx_ 100 mg, 14.9 ISIS-CRP_Rx_ 200 mg and 14.6 ISIS-CRP_Rx_ 400 mg) as was the mean number of days of treatment (81 placebo, 86 ISIS-CRP_Rx_ 100 mg, 86 ISIS-CRP_Rx_ 200 mg and 85 ISIS-CRP_Rx_ 400 mg).

### Pharmacodynamics

Robust, durable, selective and dose dependent reductions of hs-CRP were observed by Day 36, with the effects preserved at Day 92 (Table 2 and Additional file 3). There was a large placebo response (45% reduction at Day 92) observed in hs-CRP. There were no meaningful differences or trends between placebo and the ISIS-CRP_Rx_ treatment in the other markers of inflammation (that is, fibrinogen, SAA, TNF-α, cytokine IL-6, TNF-RI and TNF-RII) at either Day 36 or Day 92. Mean baseline, Day 36 and 92 results are presented in Table 2 for TNF-α, IL-6 and SAA.

**Table 2:** **Inflammation markers and disease activity outcomes (per protocol population)**

	**Placebo**	**ISIS-CRP**_**Rx**_		**ISIS-CRP**_**Rx**_	**p-value**	**ISIS-CRP**_**Rx**_	
	**Nimber = 11**	**100 mg**		**200 mg**		**400 mg**	
		**Number = 10**		**Number = 8**		**Number = 11**	
**INFLAMMATION MARKERS**			***P*** **-value** ^**a**^		***P*** **-value** ^**a**^		***P*** **-value** ^**a**^
**hs-CRP (mg/L)**	**Number = 11**	**Number = 10**		**Number = 8**		**Number = 11**	
Baseline, mean (SD)	22.4 (18.1)	29.7 (29.0)	0.97	35.2 (36.4)	0.24	19.3 (13.6)	0.65
	**Number = 10**	**Number = 10**		**Number = 8**		**Number = 11**	
Day 36, mean (SD)	16.4 (12.3)	16.9 (16.2)	0.85	19.1 (25.9)	0.57	4.26 (4.2)	0.004
Day 36, mean change from baseline (SD)	−6.8 (15.6)	−12.8 (20.9)	0.85	−16.1 (12.1)	0.15	−15.0 (11.5)	0.07
Day 36, percentage change from baseline, mean (SD)	−14.4 (66.3)	−19.5 (63.6)	0.97	−56.6 (26.2)	0.10	−76.7 (16.4)	0.002
	**Number = 11**	**Number = 10**		**Number = 8**		**Number = 11**	
Day 92, mean (SD)	10.8 (10.5)	15.0 (19.1)	0.93	12.0 (10.7)	0.89	5.6 (5.2)	0.22
Day 92, mean change from baseline (SD)	−11.6 (17.3)	−14.7 (18.9)	0.81	−23.2 (28.2)	0.13	−13.7 (13.3)	0.80
Day 92, percentage change from baseline, mean (SD)	−44.5 (60.2)	−33.8 (82.3)	0.86	−66.9 (23.0)	0.49	−64.4 (38.6)	0.19
			***P*** **-value** ^**a**^		***P*** **-value** ^**a**^		***P*** **-value** ^**a**^
**TNF-α (pg/mL)**	**Number = 11**	**Number = 10**		**Number = 8**		**Number = 11**	
Baseline, mean (SD)	15.6 (0.0)	15.6 (0.0)	1.00	15.6 (0.0)	1.00	15.6 (0.0)	1.00
	**Number = 10**	**Number = 10**		**Number = 8**		**Number = 11**	
Day 36, mean (SD)	15.6 (0.0)	15.6 (0.0)	1.00	15.6 (0.0)	1.00	15.6 (0.0)	1.00
	**Number = 11**	**Number = 10**		**Number = 8**		**Number = 11**	
Day 92, mean (SD)	15.6 (0.0)	15.6 (0.0)	1.00	15.9 (0.8)	0.42	16.0 (1.4)	1.00
			***P*** **-value** ^**a**^		***P*** **-value** ^**a**^		***P*** **-value** ^**a**^
**IL-6 (pg/mL)**	**Number = 10**	**Number = 9**		**Number = 8**		**Number = 11**	
Baseline, mean (SD)	24.6 (20.3)	46.2 (45.3)	0.24	35.7 (32.9)	0.57	25.6 (26.1)	0.92
	**Number = 10**	**Number = 10**		**Number = 7**		**Number = 11**	
Day 36, mean (SD)	21.4 (19.8)	35.7 (32.5)	0.68	27.9 (21.6)	0.67	28.3 (42.1)	0.97
	**Number = 11**	**Number = 10**		**Number = 7**		**Number = 11**	
Day 92, mean (SD)	30.3 (41.6)	32.1 (28.1)	0.65	83.1 (142.6)	0.20	53.5 (62.3)	0.30
			***P*** **-value** ^**a**^		***P*** **-value** ^**a**^		***P*** **-value** ^**a**^
**Serum Amyloid A (mg/L)**	**Number = 11**	**Number = 10**		**Number = 8**		**Number = 11**	
Baseline, mean (SD)	75.5 (167.4)	67.9 (85.4)	0.92	171.1 (255.1)	0.35	35.5 (44.4)	0.62
	**Number = 10**	**Number = 10**		**Number = 8**		**Number = 11**	
Day 36, mean (SD)	33.9 (41.2)	45.8 (38.4)	0.44	105.1 (192.2)	0.24	48.3 (86.4)	0.67
	**Number = 11**	**Number = 10**		**Number = 8**		**Number = 11**	
Day 92, mean (SD)	24.8 (30.0)	42.6 (39.2)	0.28	96.8 (112.0)	0.03	88.9 (98.9)	0.07
**DISEASE ACTIVITY OUTCOMES**							
**ACR**			***P*** **-value** ^**b**^		***P*** **-value** ^**b**^		***P*** **-value** ^**b**^
**Day 36, number (% of responders)**	**Number = 10**	**Number = 10**		**Number = 8**		**Number = 11**	
ACR20% by hs-CRP	2 (20.0)	3 (30.0)	1.000	0	0.477	4 (36.4)	0.635
ACR20% by ESR	2 (20.0)	3 (30.0)	1.000	0	0.477	3 (27.3)	1.000
ACR50 by hs-CRP	1 (10.0)	2 (20.0)	1.000	0	1.000	1 (9.1)	1.000
ACR50 by ESR	0	2 (20.0)	0.474	0		0	
			***P*** **-value** ^**b**^		***P*** **-value** ^**b**^		***P*** **-value** ^**b**^
**Day 92, number (% of responders)**	**Number = 11**	**Number = 10**		**Number = 8**		**Number = 11**	
ACR20 by hs-CRP	6 (54.5)	5 (50.0)	1.000	1 (12.5)	0.147	4 (36.4)	0.392
ACR20 by ESR	6 (54.5)	4 (40.0)	0.670	1 (12.5)	0.147	4 (36.4)	0.392
ACR50 by hs-CRP	2 (18.2)	2 (20.0)	1.000	0	0.485	3 (27.3)	1.000
ACR50 by ESR	1 (9.1)	2 (20.0)	0.586	0	1.000	2 (18.2)	1.000
**DAS28-ESR**			***P*** **-value** ^**a**^		***P*** **-value** ^**a**^		***P*** **-value** ^**a**^
	**Number = 11**	**Number = 10**		**Number = 8**		**Number = 11**	
Baseline, mean (SD)	6.7 (0.6)	6.4 (1.1)	0.4298	6.3 (0.7)	0.3352	6.2 (0.9)	0.2489
	**Number = 10**	**Number = 10**		**Number = 8**		**Number = 11**	
Day 36, mean (SD)	6.2 (1.1)	5.4 (1.5)	0.1097	5.8 (1.1)	0.4974	5.2 (1.2)	0.0572
Day 36, mean change from baseline (SD)	−0.5 (0.6)	−1.0 (0.9)	0.2176	−0.4 (0.7)	0.8286	−1.0 (0.8)	0.0986
Day 36, percentage change from baseline, mean(SD)	−8.0 (10.7)	−16.3 (16.1)	0.1431	−7.1 (11.2)	0.8968	−16.8 (12.7)	0.1321
	**Number = 11**	**Number = 10**		**Number = 8**		**Number = 11**	
Day 92, mean (SD)	5.3 (1.6)	5.2 (1.5)	0.8711	5.6 (1.1)	0.7164	5.1 (1.2)	0.6880
Day 92, mean change from baseline (SD)	−1.3 (1.2)	−1.1 (1.1)	0.6833	−0.7 (0.7)	0.2422	−1.1 (1.3)	0.6887
Day 92, percentage change from baseline, mean (SD)	−20.7 (19.7)	−17.5 (18.1)	0.6873	−11.7 (11.1)	0.2982	−16.9 (21.2)	0.6237

^a^ *P*-values calculated using a pairwise comparison between active treatment and placebo using the analysis of variance or Wilcoxon Rank Sum test; ^b^ *P*-values calculated using a pairwise comparison between active treatment and placebo using the Fisher’s Exact Test or Chi-square test. ACR, American College of Rheumatology; DAS28-ESR, disease activity score 28-erythrocyte sedimentation rate (DAS28-ESR = 0.56*square root of tender joint count + 0.28* square root of swollen joint count + 0.014*subject assessment of pain + 0.70*ln(ESR)); ESR, erythrocyte sedimentation rate; hs-CRP, high sensitivity C-reactive protein; SD, standard deviation.

### Exploratory disease activity

As compared with placebo, there were no statistically significant effects seen with ISIS-CRP_Rx_ on disease symptoms as assessed via ACR20, ACR50 or DAS28-ESR (Table 2) at Day 36 or Day 92.

### Safety

ISIS-CRP_Rx_ was generally safe and well tolerated with no serious infections due to treatment. There were similar numbers of patients who reported treatment emergent adverse events (TEAEs) across the treatment groups (8/12 placebo, 7/12 ISIS-CRP_Rx_ 100 mg, 11/13 ISIS-CRP_Rx_ 200 mg and 12/14 ISIS-CRP_Rx_ 400 mg) (Table 3). There were a similar number of infections reported in each group (Table 3). Infections were reported as mostly mild in severity with only a few reported as moderate (Table 3).

**Table 3:** **Adverse events (safety population)**

	**Placebo**	**ISIS-CRP**_**Rx**_	**ISIS-CRP**_**Rx**_	**ISIS-CRP**_**Rx**_
		**100 mg**	**200 mg**	**400 mg**
	**Number = 12**	**Number = 12**	**Number = 13**	**Number = 14**
**Overall adverse events, n (%)**	8 (66.7)	7 (58.3)	11 (84.6)	12 (85.7)
**Adverse events with incidence 10% or greater in all ISIS-CRPRx dosed patients by treatment excluding adverse events at the injection site,**				
Rheumatoid arthritis, number (%)	1 (8.3)	1 (8.3)	2 (15.4)	2 (14.3)
Blood creatinine increased, number (%)	1 (8.3)	2 (16.7)	1 (7.7)	1 (7.1)
**Adverse events-infections, number (%)**	4 (33.3)	1 (8.3)	2 (15.4)	4 (28.6)
Nasopharyngitis, number (severity)	1 (moderate)	1 (mild)	1 (mild)	1 (moderate)
Upper respiratory infection, number (severity)	0	0	0	1 (mild)
Rhinitis, number (severity)	1 (mild)	0	0	0
Bronchitis, number (severity)	2 (moderate)	0	1 (moderate)	0
Urinary tract infection/cystitis, number (severity)	0	1 (mild)	1 (moderate)	2 (mild)
Foot infection, number (severity)	0	0	0	1 (mild)
Pyelonephritis chronic, number (severity)	1 (mild)	0	0	0
**Adverse events-injection site reactions**				
Overall, number (%)	0	3 (25)	3 (23)	3 (21)
Erythema, number (%)	0	3 (25)	3 (23)	3 (21)
Pruritus, number (%)	0	1 (8)	0	0
Pain, number (%)	0	0	0	1 (7)
Swelling, number (%)	0	0	0	1 (7)

There were seven patients who discontinued treatment either due to TEAEs or meeting the requirements for one of the stopping rules. One patient on ISIS-CRP_Rx_ 200 mg discontinued treatment due to fatal pulmonary edema which also met the criteria for a serious adverse event (SAE). This patient had known atherosclerotic vascular disease. The investigator rated this as possibly related to study drug. Two patients discontinued treatment due to worsening of RA symptoms. One was on ISIS-CRP_Rx_ 200 mg and the other was on ISIS-CRP_Rx_ 400 mg. Three patients (one placebo, one ISIS-CRP_Rx_ 200 mg and one ISIS-CRP_Rx_ 400 mg) met the protocol pre-specified stopping criteria for renal function (that is, serum creatinine (Cr) increase >0.2 mg/dL). However, the absolute serum Cr levels remained within the normal limits for all of these patients. One patient on ISIS-CRP_Rx_ 200 mg discontinued treatment due to an exacerbation of chronic calculous cholecystitis although this was considered unlikely related to study drug by the investigator.

## Discussion

ISIS 329993 (ISIS-CRP_Rx_) is a novel approach to reducing the inflammatory marker CRP and to potentially down regulate inflammation. As an antisense drug targeting human CRP it was hypothesized that ISIS-CRP_Rx_ should be non-immunosuppressant and it, therefore, might be a valuable adjunct to standard DMARDs, or even biologic DMARDs. Previous studies of ISIS-CRP_Rx_ in rodent models of RA (that is, CRP transgenic mice with collagen-induced arthritis) demonstrated improvement in the clinical signs of arthritis [4]. Subsequently, initial clinical studies in healthy human volunteers demonstrated that treatment with ISIS-CRP_Rx_ achieved significant hs-CRP lowering [4]. Before embarking on a large RA study to definitively address the question of clinical improvement in symptoms and signs, it was decided to first explore whether this approach would reproducibly decrease hs-CRP in RA patients and to assess its potential impact on other markers of inflammation. Given the results of this study, ISIS-CRP_Rx_ has demonstrated that the antisense oligonucleotide platform can selectively reduce circulating levels of hs-CRP. The results at 36 days demonstrate separation between dosing cohorts with respect to percent change in hs-CRP and achieved statistical significance at the highest dose, with a 76.7% reduction in hs-CRP in the ISIS CRP_Rx_ 400 mg arm compared with a 14.4% reduction in the placebo arm at Day 36 (*P* = 0.0015). However, by 92 days this response does not appear as statistically robust given the significant decreases in hs-CRP seen in the placebo group. It is unclear what drove the decreases in hs-CRP in the placebo group but it could be speculated that it was in part regression to the mean, and possibly related to increased compliance with background regimens that can also be seen in clinical trials as a consequence of closer follow-up.

It remains unclear whether ISIS-CRP_Rx_ is truly anti-inflammatory in an active human disease state. Despite the encouraging data in the animal models, there was little change in the other inflammatory markers studied in this trial including ESR, SAA, IL-6, TNF-α, fibrinogen and complement. This invites the question as to whether inflammation was down regulated or whether the drug was simply effective at blocking a surrogate marker of inflammation. Additionally, the exploratory endpoints of ACR20/50 and DAS28-ESR were frankly difficult to interpret in the setting of such small numbers and such high placebo response rates. That said, the reductions observed in the clinical measures of disease activity were not of sufficient magnitude to offer significant benefit over the currently available drugs for treatment of RA.

It appeared that ISIS-CRP_Rx_ was well tolerated in a population with active autoimmune/inflammatory disease. However, the small sample size limits the ability to detect uncommon safety signals.

There were some limitations to this study, most notably the overall size and powering together with the very large placebo response rate limited the conclusions that could be drawn. However, even with a small sample size, the lack of any discernible effect on other markers of inflammation coupled with the very modest clinical changes make it unlikely that ISIS-CRP_Rx_ will be an adequately effective drug for treatment of rheumatoid arthritis when compared with other currently available and experimental therapies.

## Conclusions

In this study, ISIS-CRP_Rx_ selectively reduced hs-CRP in a dose-dependent manner, and was well-tolerated in patients with RA. Its utility as a therapy in RA remains unclear. There may be other chronic inflammatory conditions in which CRP has a causative or contributory role; so additional studies in other diseases may be warranted given the tolerability of ISIS-CRP_Rx_, the lack of obvious immune suppression and the potential for combination with other agents.

## Additional files

Additional file 1:
**Listing of Ethics Committees and Institutional Review Boards (IRB).**
Additional file 2:
**Flow of patients through study. CONSORT patient disposition diagram.**
Additional file 3:
**Change in hs-CRP by treatment group.** Panel A shows mean absolute change from baseline (mg/L) in hs-CRP at Day 36 and Day 92 by dose group. Panel B shows mean percent change from baseline in hs-CRP at Day 36 and Day 92 by dose group).

## Abbreviations

ACR20: American College of Rheumatology 20% improvement criteria
ACR50: American College of Rheumatology 50% improvement criteria
Cr: creatinine
CRP: C-reactive protein
DAS28-ESR: Disease Activity Score Activity Score Calculator for Rheumatoid Arthritis using the ESR
DMARD: disease-modifying antirheumatic drugs
ESR: erythrocyte sedimentation rate
Hs-CRP: high sensitivity C-reactive protein assay
NSAIDs: non-steroidal anti-inflammatory drugs
RA: rheumatoid arthritis
SAA: serum amyloid A
TEAEs: treatmemt emergent adverse effects
TNF-R2: tumor necrosis factor receptor 2
TNF-RI: tumor necrosis factor receptor 1
TNF-α: tumor necrosis factor alpha

## References

[1] Emery P, Gabay C, Kraan M, Gomez-Reino J (2007). Evidence-based review of biologic markers as indicators of disease progression and remission in rheumatoid arthritis. Rheumatol Int.

[2] Molenaar ET, Voskuyl AE, Familian A, van Mierlo GJ, Dijkmans BA, Hack CE (2001). Complement activation in patients with rheumatoid arthritis mediated in part by C-reactive protein. Arthritis Rheum.

[3] Dessein PH, Joffe BI, Stanwix AE (2004). High sensitivity C-reactive protein as a disease activity marker in rheumatoid arthritis. J Rheumatol.

[4] Jones NR, Pegues MA, McCrory MA, Singleton W, Bethune C, Baker BF (2012). A selective inhibitor of human C-reactive protein translation is efficacious in vitro and in C-reactive protein transgenic mice and humans. Mol Ther Nucleic Acids.

[5] Arnett FC, Edworthy SM, Bloch DA, McShane DJ, Fries JF, Cooper NS (1988). The American Rheumatism Association 1987 revised criteria for the classification of rheumatoid arthritis. Arthritis Rheum.

[6] Hochberg MC, Chang RW, Dwosh I, Lindsey S, Pincus T, Wolfe F (1992). The American College of Rheumatology 1991 revised criteria for the classification of global functional status in rheumatoid arthritis. Arthritis Rheum.

[7] Felson DT, Anderson JJ, Boers M, Bombardier C, Furst D, Goldsmith C (1995). American College of Rheumatology. Preliminary definition of improvement in rheumatoid arthritis. Arthritis Rheum.

[8] Prevoo ML, Van ‘t Hof MA, Kuper HH, van Leeuwen MA, van de Putte LB, van Riel PL (1995). Modified disease activity scores that include twenty-eight-joint counts. Development and validation in a prospective longitudinal study of patients with rheumatoid arthritis. Arthritis Rheum.

[9] ISIS Pharmaceuticals Inc. Data on File. \\isis.local\ogroups\cdmstats\329993\CS1\testdir\CohortIII\programs\thsCRPetCIII.sas DCHEN SASv9.2 (23MAR2011 14:13) Data Download: 21 March 2011.

