# Supplementary material for: Results of a proof of concept, double-blind, randomized trial of a second generation antisense oligonucleotide targeting high-sensitivity C-reactive protein (hs-CRP) in rheumatoid arthritis
Source: Arthritis Res Ther. 2015 Mar 19;17(1):80. doi: 10.1186/s13075-015-0578-5 (PMC4415222; doi:10.1186/s13075-015-0578-5)
Supplement: Additional file 1: — Listing of Ethics Committees and Institutional Review Boards (IRB). [file 13075_2015_578_MOESM1_ESM.doc]

# Appendix I

Listing of Ethics Committees and Institutional Review Boards (IRB)

| IRB |
| --- |
| IRB Services 372 Hollandview Trail, Suite 300 Aurora, ON Canada L4G 0A5 |
| Ethical Council Affiliated to Ministry of Healthcare and Social Development of Russian Federation  3 Rakhmanovsky pereulok,  Moscow 127994, Russian Federation  Ethical Commitee Affiliated to State Budgetary Higher Educational Institution: "St.. Petersburg State Medical University n.a. I.P Pavlov" under Ministry of Healthcare and Social Development of the Russian Federation  6/8 Lva Tolstogo Str.,  Saint Petersburg, 197022, Russian Federation  Local Ethical Committee Affiliated to Saint-Petersburg State Budgetary Healthcare Institution "City Hospital #26”  2, Kostyshko street,  Saint-Petersburg, 196247, Russian Federation  Ethical Commitee Affiliated to Federal State Budgetary Institution "Scientific Research Institution of Rheumatology" RAMN  34 A, Kashirskoe shosse,  Moscow, 115522, Russian Federation  Local Ethical Committee Affiliated to Saint-Petersburg State Budgetary Healthcare Institution “City Mariinskiy Hospital”  56, Liteiniy prospect,  Saint-Petersburg, 191104, Russian Federation  Ethics Committee Affiliated to State Budgetary Educational Institution of Higher Professional Education "Moscow State Medico-Stomatological University of Ministry of Health and Social Development of Russian Federation"  20 bld.1, Delegatskaya street,  Moscow, 127473 Russian Federation  Local Ethics Committee Affiliated to Saint-Petersburg Budgetary State Healthcare Institution “City Pokrovskaya Hospital”  85, V.O. Bolshoy prospekt  Saint-Petersburg, 199106, Russian Federation  Local Ethics Committee Affiliated to State Budgetary Healthcare Institution “Ryazan Regional Clinical Cardiology Dispensary”  96, Stroykova str.  Ryazan, 390026, Russian Federation  Local Ethics Committee Affiliated to Almazov Federal Heart, Blood and Endocrinology Centre of Department of Health and Social Development of Russian Federation  2, Akkuratova str.  Saint Petersburg, 197341, Russian Federation  Local Ethics Committee Affiliated to State Budgetary Healthcare Institution of Moscow City Clinical Hospital #1 n.a. N.I. Pirogov of Moscow Healthcare Department  8, Leninskiy pr.  Moscow, 119049, Russian Federation |
