# Supplementary figures and images for: Results of a proof of concept, double-blind, randomized trial of a second generation antisense oligonucleotide targeting high-sensitivity C-reactive protein (hs-CRP) in rheumatoid arthritis
Source: Arthritis Res Ther. 2015 Mar 19;17(1):80. doi: 10.1186/s13075-015-0578-5 (PMC4415222; doi:10.1186/s13075-015-0578-5)

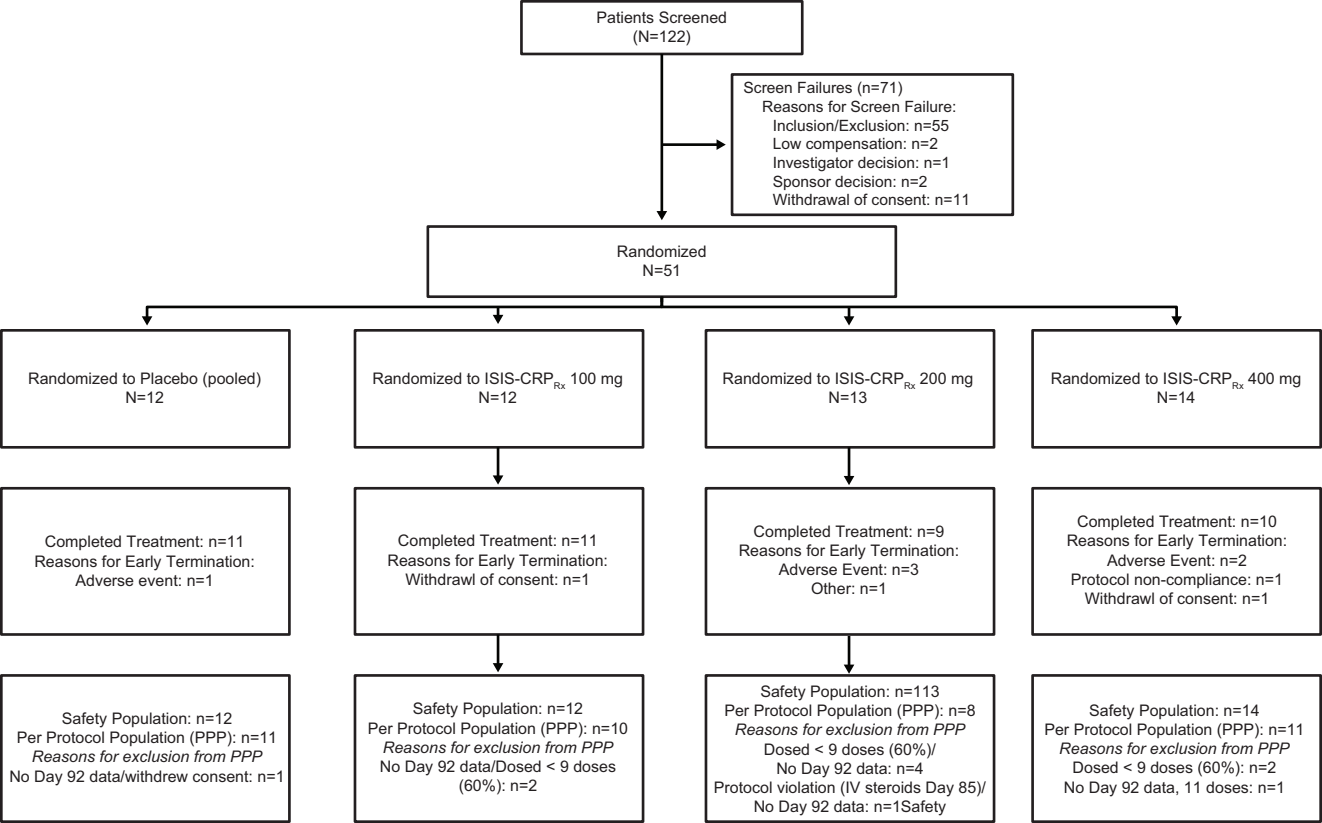

Supplement: Additional file 2: — Flow of patients through study. CONSORT patient disposition diagram. [file 13075_2015_578_MOESM2_ESM.pdf]

**A**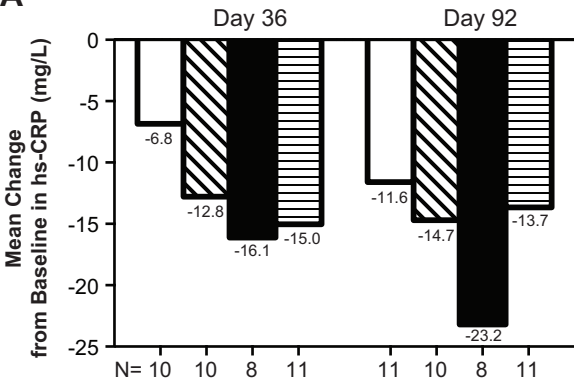**B**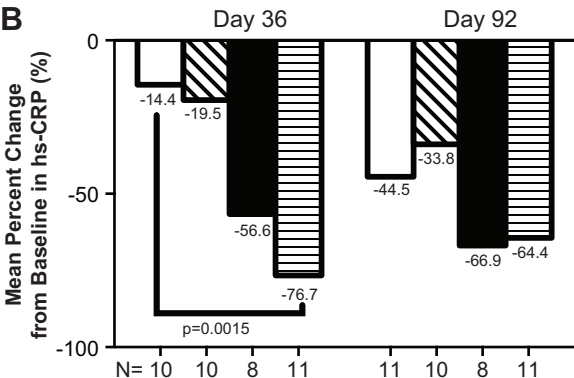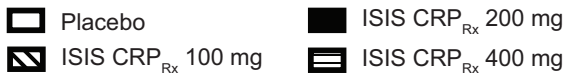

Supplement: Additional file 3: — Change in hs-CRP by treatment group. Panel A shows mean absolute change from baseline (mg/L) in hs-CRP at Day 36 and Day 92 by dose group. Panel B shows mean percent change from baseline in hs-CRP at Day 36 and Day 92 by dose group). [file 13075_2015_578_MOESM3_ESM.pdf]
